# Exchangeable and Plant-Available Macronutrients in a Long-Term Tillage and Crop Rotation Experiment after 15 Years

**DOI:** 10.3390/plants11040565

**Published:** 2022-02-21

**Authors:** Reinhard W. Neugschwandtner, Jiřina Száková, Vera Pachtrog, Pavel Tlustoš, Martin Kulhánek, Jindřich Černý, Hans-Peter Kaul, Helmut Wagentristl, Gerhard Moitzi, Pia Euteneuer

**Affiliations:** 1 Institute of Agronomy, Department of Crop Sciences, University of Natural Resources and Life Sciences Vienna (BOKU), Konrad-Lorenz-Straße 24, 3430 Tulln, Austria; pachtrog@yahoo.com (V.P.); hans-peter.kaul@boku.ac.at (H.-P.K.); 2 Department of Agro-Environmental Chemistry and Plant Nutrition, Faculty of Agrobiology, Food and Natural Resources, Czech University of Life Sciences Prague, Kamýcká 129, 165 21 Prague, Czech Republic; szakova@af.czu.cz (J.S.); tlustos@af.czu.cz (P.T.); kulhanek@af.czu.cz (M.K.); cernyj@af.czu.cz (J.Č.); 3 Experimental Farm Groß-Enzersdorf, Department of Crop Sciences, University of Natural Resources and Life Sciences Vienna (BOKU), Schloßhoferstraße 31, 2301 Groß-Enzersdorf, Austria; helmut.wagentristl@boku.ac.at (H.W.); gerhard.moitzi@boku.ac.at (G.M.); pia.euteneuer@boku.ac.at (P.E.)

**Keywords:** phosphorus, potassium, sulphur, calcium, magnesium

## Abstract

The status of macronutrients phosphorus (P), potassium (K), sulphur (S), calcium (Ca) and magnesium (Mg) was assessed 15 years after the establishment of a long-term crop rotation and soil tillage trial with mouldboard ploughing (MP), no-till (NT), deep conservation tillage (CTd) and shallow conservation tillage (CTs). The mobile proportions of macronutrients in an Austrian Chernozem soil were determined to a depth of 50 cm with the single reagent extractant acetic acid (AA) and Mehlich 3 (M3), which uses several reagents as extractants. AA revealed less P and K, but more Ca and Mg compared to M3. Both extractants could capture the distribution pattern of the nutrients in the soil profile, but M3 showed higher differences among the soil layers. In the first 5 cm in NT, the P concentration was higher than in MP, CTd and CTs. The concentration of K was higher in NT, CTd and CTs than in MP in the first 10 cm of the soil. Phosphorus and K concentrations did not differ between tillage treatments below these soil layers, and S, Ca and Mg were similar in all soil layers. As none of the analysed elements except for Ca were fertilized and no accumulation of S, Ca and Mg was observed in the upper soil layer, the higher concentrations are attributed to accumulation through crop residues and then less leaching of P and K. Crop rotation did not affect the distribution of the analysed macronutrients in the soil but affected the nutrient uptake by winter wheat mostly due to the yield differences of winter wheat in the two crop rotations.

## 1. Introduction

Tillage systems are generally categorized into conventional tillage, where the soil is inverted by using a mouldboard plough, conservation tillage, where the soil is cultivated by using a chisel plough, disk plough, harrow disk or cultivators but not inverted, and no-till, where seeds are sown directly into the untilled soil.

Conventional tillage is being increasingly superseded by reduced tillage systems on a global perspective [1]. Yields of no-till were within a five percent range of those obtained by mouldboard ploughing in a review on crop yield from several European countries [2]. Winter wheat (*Triticum aestivum* L.) yields in Eastern Austria were generally at similar levels in conventional, conservation and no-till, but no-till has higher yields in very dry years, and conventional and conservation tillage have higher ones with higher amounts of rainfall during the growth period [3].

No-till has several economic benefits: fuel consumption and working time for establishing winter wheat can be reduced compared to conventional tillage by about 85% [4,5], and the direct energy input is lower as shown for winter wheat [6], sugar beet and soybean [7] and maize; in maize, no-till also resulted in the highest energy efficiency [8]. No-till enhances the biotic activity, e.g., of earthworms, and reduces soil erosion [1]. Soil chemical properties are altered by soil tillage. In a previous study conducted in the same field experiment, electrical conductivity, cation exchange capacity, total organic carbon and total nitrogen were higher in the upper soil layer of no-till compared to conventional tillage after 15 years, whereas pH_CaCl2_ and pH_H2O_ did not change [9].

In the soil, elements are present in different chemical forms which influence their bioavailability. There are various methods for an estimation of the element bounds in the individual soil fractions by using so-called operationally defined extraction procedures. Within these methods, there are either single extractions used predominantly for the assessment of the bioavailable proportions of elements, or sequential extraction procedures estimating the element bounds on the individual soil fractions. For instance, elements can be categorized according to their extractability by reagents, according to Tessier et al. [10], into the following fractions: (1) “exchangeable”, (2) “bound to carbonates”, (3) “bound to iron and manganese oxides”, (4) “bound to organic matter” and (5) “residual”. Tack and Verloo [11] refer to fractions 2–4 as (2) “acid extractable”, (3) “reducible” and (4) “oxidisable”.

Acetic acid (AA) is a single reagent extractant. Diluted AA releases exchangeable, water and acid soluble element fractions. It is mainly used for the assessment of trace elements [12,13], and it dissolves a wide range of minerals due to its lack of selectivity [14]. The Mehlich 3 (M3) soil test is a multi-nutrient soil test suitable for a wide range of soils which uses several reagents as extractants [15]. It is used for macro- and micronutrients and because of the combined several extraction mechanisms [16].

The aim of this study was to assess the influence of four different soil tillage systems and two crop rotations on the distribution of bioavailable proportions of macronutrients in soil layers down to 50 cm depth after fifteen years since the establishment of the field trial on Chernozem in East Austria. Two extraction methods were used and compared: extraction with AA or M3 to assess their applicability for the estimation of the bioavailable pool of nutrients. Acetic acid is used for the fast screening of the mobilizable pool of chemical elements in soils and sediments [17]. Therefore, the use of AA could simplify the analytical procedure of the determination of the bioavailable pool of nutrients in soils compared to M3 extractable nutrients.

## 2. Materials and Methods

### 2.1. Experimental Site and Experimental Design

The long-term experiment was established in 1996 in Raasdorf (48°14′ N, 16°33′ E; altitude: 153 m a.s.l) in East Austria at the experimental farm of the University of Natural Resources and Life Sciences, Vienna (BOKU). Raasdorf is located on the edge of the Marchfeld plain, an important crop production region in the north-western part of the Pannonian Basin. The silty loam Chernozem is of alluvial origin and is rich in calcareous sediments. The mean annual temperature is 10.7 °C, and the mean annual precipitation is 543 mm (1983–2012).

The split-plot design with four replication blocks involves two factors: tillage (main plots, 24 × 40 m) and crop rotation (subplots, 12 × 40 m). Tillage variants are: (I) Mouldboard ploughing (MP) after harvest to a soil depth of 25–30 cm. The loosened soil is inverted, and thereby, residues are fully incorporated into the soil. (II) No-till (NT): Direct drilling in un-tilled soil with a disc drill without previous removal of residues. A total herbicide is sprayed before sowing for weed control. (III) Deep conservation tillage (CTd) to a soil depth of 20–25 cm using a wing share cultivator and, every four years, a subsoiler is used to a depth of 35 cm. A part of the plant residue remains on the soil surface. (IV) Shallow conservation tillage (CTs) to a soil depth of 8–10 cm using a wing share cultivator. A high share of the plant residue remains on the soil surface. Two variable crop rotations are performed on sub-plots. Both rotations frequently included winter wheat; besides that, the central crops are sugar beet (crop rotation A; CR A) or maize (crop rotation B; CR B). Crop protection and nitrogen (N) fertilization was performed crop-specific according to good agricultural practice. The N fertilizer used was mostly calcium ammonium nitrate (27% N, 10% Ca). No other elements except for N and Ca were applied. For further details, see Neugschwandtner et al. [3].

### 2.2. Soil Sampling and Sample Preparation

Soil sampling was performed in 5 cm steps at a depth of 0–30 cm, and in 10 cm steps at a depth of 30–50 cm from 7 to 9 November 2011, with soil probes (Purckhauer type, core diameter: 18 mm). A composite sample of 30 equally sized, discrete sub-samples randomly collected was composed per plot for each sampled layer. Air-dried samples were homogenized and sieved (2 mm). Crops grown before sampling had been harvested in July (winter wheat) and in October (sugar beet).

### 2.3. Soil Analysis

The exchangeable (mild acid extractable) element portions were determined by extracting the soil samples with 0.11 mol L^−1^ acetic acid (CH_3_COOH) at a ratio of 1:20 (*w*/*v*) for 16 h [18,19]. The extracts were then centrifuged at 3000 rpm for 10 min; the supernatants were kept at 6 °C prior to measurements.

The plant-available element portions were determined by extraction with Mehlich 3 (0.2 mol L^−1^ CH_3_COOH + 0.25 mol L^−1^ ammonium nitrate (NH_4_NO_3_) + 0.013 mol L^−1^ nitric acid (HNO_3_) + 0.015 mol L^−1^ ammonium fluoride (NH_4_F) + 0.001 mol L^−1^ ethylene diamine tetra-acetic acid (EDTA)) at a ratio of 1:10 (*w*/*v*) for 10 min followed by filtration [15].

The five extractants combined different extraction mechanisms: exchangeable elements are extracted by acetic acid [19] and ammonium nitrate [20]. Nitric acid partly destroys organic matter and oxidizes sulphide compounds [21]. Ammonium fluoride displaces with NH_4_^+^ the metal cations Ca, K and Mg from exchange sites on soil colloids [22], and the fluoride ion can complex Al and Fe which releases P from Al- and Fe-phosphates [23,24]. EDTA is a chelating agent, which proved to be the most effective among several tested for Pb desorption from soils [25]. It is tested for the enhanced phytoremediation of heavy-metal contaminated soil [26,27] but also co-mobilizes unspecifically macro- and micronutrients, including negatively charged P [28,29].

The soil extracts were analysed using inductively coupled plasma optical emission spectrometry (ICP–OES, Varian, VistaPro, Australia) for P and S and flame atomic absorption spectroscopy (FAAS, VARIAN SpectrAA-280, Victoria, Australia) for Ca, K and Mg. The certified reference material silty clay loam (Analytika Ltd., Praha, Czech Republic) was used for quality assurance of the analytical data.

### 2.4. Winter Wheat Cultivation and Sampling

The winter wheat (cv. Astardo) was planted in October and harvested on 5 July 2012. The harvest index and the nutrient harvest index were calculated as the percentage of grain or nutrients in the grain on the above-ground dry matter or the amount of nutrients in the above-ground dry matter, respectively. Yields of winter wheat in 2012 were considerably below the long-term average due to a severe drought during the growing period, and thereby, the lowest ones among 12 years when winter wheat was grown in that long-term experiment [3].

The total concentrations of elements in grain and straw were determined in the digests obtained by the following decomposition procedure: 0.5 g of the dried and powdered plant matter was decomposed in a digestion vessel with a mixture of 8 mL concentrated nitric acid and 2 mL of hydrogen peroxide. The mixture was heated in an Ethos 1 (MLS GmbH, Leutkirch im Allgäu, Germany) microwave-assisted wet digestion system for 33 min at 210 °C. The digest was then transferred into a 20 mL glass tube, filled with deionised water, and kept at laboratory temperature until measurement. Plant digests were analysed using ICP–OES (for P and S) and FAAS (for Ca, K and Mg). Certified reference material NCS DC 73,348 Bush Branches and Leaves was used for quality assurance of analytical data.

### 2.5. Statistical Analysis

Analysis of variance for soil macronutrients (PROC MIXED) and plant parameters (PROC GLM) was performed using software SAS version 9.2. Means were separated by least significant differences (LSD) when the F-test indicated factorial effects on the significance level of *p* < 0.05. Based on the results, data are presented as main effects of depth or as tillage × depth interactions (means over both crop rotations). As no significant differences were observed for crop rotation, means are shown for each rotation (over all depths and tillage treatments).

## 3. Results

### 3.1. Macronutrients in the Soil Profile

**Figure 1:**
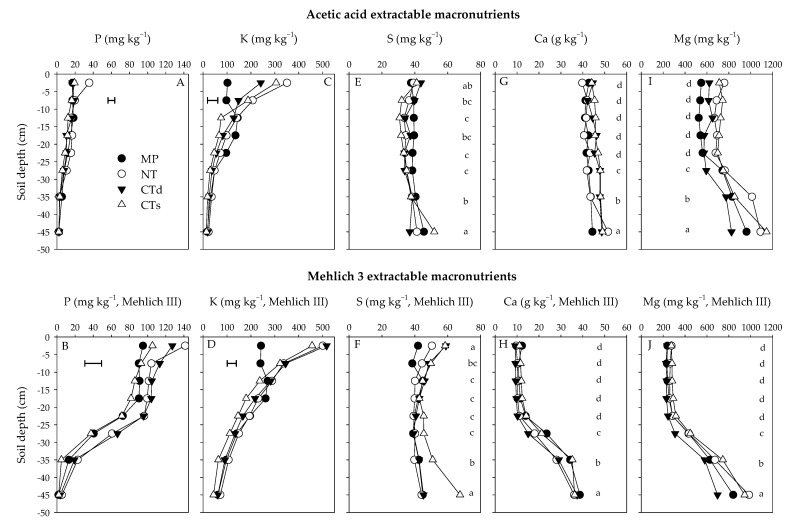
Acetic acid extractable and Mehlich 3 extractable macronutrients (**A**,**B**) phosphorus (P), (**C**,**D**) potassium (K), (**E**,**F**) sulphur (S), (**G**,**H**) calcium (Ca) and (**I**,**J**) magnesium (Mg) at a soil depth of 0–50 cm after 15 years of different tillage treatments in Raasdorf (Austria) in November 2011: mouldboard ploughing (MP), no-till (NT), deep conservation tillage (CTd) and shallow conservation tillage (CTs). Different letters having no letter in common are significantly different between the soil layers (main effects, *p* < 0.05). Horizontal bars indicate significant tillage × depth interactions (LSD, *p* < 0.05). Mean concentrations over two crop rotations.

Phosphorus was, over all tillage treatments, highest in the uppermost soil layers and decreased gradually. There was a significant tillage × depth interaction. In the uppermost soil layer, AA-extracted P was ranked as follows: NT > CTs, MP, CTd, and the M3-extracted P as follows: NT ≥ CTd ≥ CTs ≥ MP. Further, M3-extraced P in the second soil layer was ranked as follows: CTd ≥ NT ≥ CTs, MP. No differences between tillage treatments were observed for AA-extractable P down from 5 cm and for M3-extractable P down from 10 cm soil depth (Figure 1A,B). The mean P concentrations over all tillage treatments were in the uppermost soil layer 10.5-fold (AA) or 37.4-fold (M3) higher than in the deepest soil layer. The mean concentration over all treatments and depths was 5.9-fold higher for M3 with 70.5 mg kg^−1^ compared to AA with 11.9 mg kg^−1^.

Potassium was, with both extraction procedures, highest for NT, CTd and CTs in the two uppermost soil layers and decreased gradually downwards. Concentrations of nutrients in MP were similar up to 25 (AA) or 20 (M3) cm. The main effect soil depth was ranked as follows for AA in 0-5 cm: NT > CTs > CTd > MP, in 5–10 cm: NT ≥ CTs ≥ CTd > MP, in 10–15 cm: MP, NT, CTd > CTs, in 15–20 cm: MP ≥ NT ≥ CTd, CTs, and in 20–25 cm: MP ≥ NT, CTd ≥ CTs; for M3 in 0–5 cm, it was: CTd, NT > CTs > MP, in 5–10 cm: CTd, NT, CTs > MP, in 10–15 cm: NT, CTd, MP > CTs, and in 15–25 cm: MP, NT ≥ CTd ≥ CTs (Figure 1C,D). The mean K concentrations over all tillage treatments were in the uppermost soil layer 13.6-fold (AA) or 7.2-fold (M3) higher than in the deepest soil layer. The mean concentration over all treatments and depths was 2.2-fold higher for M3 with 211 mg kg^−1^ compared to AA with 98 mg kg^−1^.

Sulphur was, with both extraction procedures, highest in the uppermost and the lowest soil layer. In any case, differences between soil layers were statistically significant, but much smaller than for other elements. Tillage did not affect the S concentrations (Figure 1E,F). The mean S concentration over all treatments and depths was 1.2-fold higher for M3 with 45.0 mg kg^−1^ compared to AA with 37.6 mg kg^−1^.

Calcium was, with both extraction procedures, lowest in the deepest soil layer and ranked as follows: 40–50 cm > 30–40 cm > 25–30 cm > 0–25 cm. The Ca concentrations did not differ between tillage treatments (Figure 1G,H). The mean Ca concentrations over all tillage treatments were, in the deepest soil layer, 1.1-fold (AA) or 3.5-fold (M3) higher than in the uppermost soil layer. The mean concentration over all treatments and depths was 2.5-fold higher for AA with 44.7 g kg^−1^ compared to M3 with 17.9 g kg^−1^.

Magnesium was, with both extraction procedures, lowest in the deepest soil layer and ranked for soil layers as follows: 40–50 cm > 30–40 cm > 25–30 cm > 0–25 cm. The Mg values did not differ between tillage treatments (Figure 1I,J). The mean Mg concentrations over all tillage treatments were, in the deepest soil layer, 1.3-fold (AA) or 3.4-fold (M3) higher than in the uppermost soil layer. The mean concentration over all treatments and depths was 2.1-fold higher for AA with 868 mg kg^−1^ compared to M3 with 404 mg kg^−1^.

Crop rotation did not affect the concentrations of AA and M3 extractable macronutrients of P, K, S, Ca and Mg. Mean concentrations for both crop rotations over the soil depth of 0–50 cm and over all four soil tillage treatments are shown in Table 1.

### 3.2. Yields, Concentrations and Uptake of Macronutrients by Winter Wheat

The grain and straw yield of winter wheat was highest with NT and lowest with MP, and higher in CR A than in CR B in 2012. The harvest index was not affected by soil tillage but was higher in CR B than CR A (Table 2).

Soil tillage did not affect the concentrations of P, K, S and Mg in the grain of winter wheat, but Ca was highest with CTs and MP and lowest with NT. The grain concentrations of P, S and Mg were higher in CR A and that of Ca higher in CR B, whereas the concentration of K was not affected by pre-crops. Soil tillage did not affect the concentrations of P, S and Mg in the straw of winter wheat, but the concentration of K was highest with NT and lowest with MP and CTs and that of Ca was lowest with NT. The concentration of P in the straw was higher in CR A than in CR B, whereas the concentrations of K, S, Ca and Mg were not affected by crop rotation.

The nutrient uptake of P, K, S and Mg in wheat grain was highest with NT and lowest with MP, and for K and Ca also with CTd. Ca uptake of winter wheat grain was highest with CTs and lowest with CTd and MP. The nutrient uptake of P, K and S in the straw of winter wheat was highest with NT and lowest with MP, and also CTs for P and CTd and CTs for K. The uptake of Ca and Mg in the straw was not affected by soil tillage. Both the grain and the straw uptake of all macronutrients was higher in CR A than in CR B.

The nutrient harvest indices of all macronutrients were not affected by soil tillage. The nutrient harvest index of Ca was higher in CR B than in CR A, whereas the indices of P, K, S and Mg were not affected by crop rotation.

## 4. Discussion

Phosphorus and K decreased, and Ca and Mg increased with soil depth. After 15 years, a depth gradient was also observed for pH_CaCl2_, pH_H2O_ and total carbon which increased with soil depth, while electrical conductivity, cation exchange capacity, total nitrogen and total organic carbon (TOC) decreased with soil depth [9]. Exchangeable fractions of K and P decrease but Mg increase with soil depth [30].

Differences between tillage treatments were observed with both extractants for P and K. The AA extractable P was higher in NT, and the M3 extractable P in NT and CTd in 0–5 cm soil depth. With both AA and M3, K was higher in NT, CTd and CTs than in MP in 0–10 cm soil depth. Significant tillage × depth interactions for P and K had already been reported seven years after the start of the experiment [31]. Similar to our observations, Franzluebbers and Hons [32] reported, for silty clay loam soil in the humid region of the south-eastern USA after 8.5 years, higher concentrations of P and K as well as Zn and Mn in NT compared to MP in the first 5 cm of the soil, but only few changes for the distribution of extractable S, Ca and Mg as well as Na in the soil profile. Liebhard [33] observed in Austria after 10 years that P tended to increase, and K significantly increased in the upper soil layer after 10 years as removal by plants was lower than the input of these nutrients with fertilization.

Two reasons are given to explain the higher nutrient concentrations in the uppermost soil layer: accumulation through residues and accumulation through unused fertilizers. For loess Chernozem in Germany, the higher soluble P (by 24%) and K (by 118%) concentrations in 0–15 cm soil depth after 16 years of conservation tillage, compared to conventional tillage, were attributed to the relocation of P and especially of K from deeper soil layers by plant residues, which contain much higher amounts of K than P [34]. Whereas the comparison of NT to MP on an Eutric Cambisol in Switzerland showed higher K and Mg concentrations for NT in 0–5 cm soil depth, but a rather uniform distribution of P and Ca for NT and MP in 0–30 cm soil depth. The higher K and Mg concentrations in the uppermost soil layer of NT but no differences for P and Ca were attributed to the fertilization regime, as K and Mg were applied at normal rates, but low amounts of P and Ca were applied. Further, the higher K and Mg concentrations were associated with the crop residue retention on the surface and a reduced plant uptake due to low pH [35]. Additionally, for a Chromic Luvisol in Pennsylvania, after 25 years higher concentrations of the elements P, K and Ca, which were applied as fertilizers, were reported in 0–15 cm soil depth, whereas no difference was reported for the not-applied Mg [36]. As only nitrogen fertilizers (which sometimes contained Ca) were applied to our experiment over the years, the higher concentrations of P up to 5 cm and K up to 10 cm soil depth in NT could only arise from the accumulation of nutrients through residues. Under Pannonian conditions, high amounts of Ca and K but low amounts of Mg and P are left with crop residues at the field after harvest as shown with chickpea, pea, barley and oat in another experiment on that location [37,38]. Consequently, only the higher amounts of P and K with NT might arise from less leaching of these elements. In lysimeter experiments, the losses of Ca and Mg were much higher than those of P and K [39] or those of K [40].

The elements P and K were more strongly extracted by M3, as were Ca and Mg by AA, whereas S was slightly strongly extracted by M3. Both M3 and AA showed the decrease of P and K and the increase of Ca and Mg down the soil profile; overall, the delta was much higher for all four elements with M3. Similarly, Száková et al. [41] reported different extractability of micronutrients as affected by soil characteristics; moreover, the differences in the extractability of elements with the individual extractants were observed. For instance, copper and iron were more easily extractable with AA compared to M3, whereas higher extractable proportions of manganese were determined for M3 compared to AA. Thus, the effectivity of the individual extractants to release the individual nutrients are affected by various factors including soil parameters, extraction agent used and the chemical properties of the released element itself.

Elements which are bound to several soil fractions, such as P and K, were more strongly extracted by M3 than by AA, as M3 combines five extractants with different extraction mechanisms. Naturally occurring P has a low solubility and a high absorption to soil and particles [42]. P is mainly bound in inorganic phosphates and in organic compounds. The share of organic P in total P ranges between 25–65% [30]. The concentration of non-exchangeable K in soils is considerably higher than available K as shown for Czech soils [43]. Long-term fertilization with farmyard manure and mineral fertilizer affect the M3 extractable concentrations of P, K, Ca and Mg in the soil, as shown in long-term experiments in the Czech Republic [44]. In these experiments, the bioavailable P concentration increased an application of organic fertilizers and was not affected by mineral fertilizer [45], and the bioavailable K increased with both organic and mineral fertilizers [46], whereas AA could better release Ca and Mg. Ca is bound in soils in easy soluble minerals, such as calcium carbonate. In Central European soils with pH values > 7, the share of Ca in exchangeable cations is >80%. Additionally, Mg in the soil solution is mainly determined by the exchangeable fractions which increases with the proportion of clay and silt [30]. The AA extractable fraction of P, K, Ca and Mg can be strongly affected by organic and inorganic amendments [47].

Diverse results have been reported in the literature for the effects of rotation in long-term tillage experiments. Total and Olsen extractable P differed in the first 20 cm of Utisol in Chile after four years of different tillage, whereas crop rotation (oat-wheat versus lupine-wheat) had no effect on P concentrations [48]. Compared to MP, NT reduced the availability of Ca and Mg but not of P and K, whereas no differences have been found for these nutrients between different crop rotations including corn, soybean and wheat on loam soil in Indiana [49]. However, in a field experiment containing the same crops in Alabama, the pH decreased in crop rotations where N fertilizer was applied, as did the P, Ca and Mg availability at lower pH values [50]. However, in Missouri, Ca and S were affected by tillage and rotation, and P and K by tillage only, whereas Mg was unaffected by both. Rotation effects on nutrient distribution were also attributed to pH changes resulting from N fertilization [51]. In our experiment, no pH changes were caused by either tillage or crop rotation [9], and no differences were found for P, K, S, Ca and Mg between crop rotation, which both received nitrogen fertilization (and partly Ca) but no other nutrients [3,8].

Crop rotation affected the concentrations of P, S, Ca and Mg (but not that of K) in the grain and of P in the straw. The tillage effect was less distinct. Consequently, the macronutrient uptake was mainly affected by CR. As the macronutrient concentrations in the soil did not differ, the different concentrations of some macronutrients in the grain and straw might result from a difference in biomass. In a different trial on this side, a nitrogen dilution effect was observed for facultative wheat, where the grain yield was higher, but the nitrogen concentration was lower with autumn sowing, compared to spring sowing [52]. In contrast, in the current experiment, only Ca in the grain was higher with a lower grain yield, although other affected nutrient concentrations were also higher in the rotation where the higher yields occurred. No statistically significant correlation for P, Ca and Mg and just a week correlation (*p* < 0.10) for K was reported for AA extractable soil concentrations and element concentrations in maize leaves [53]. Whereas the phosphorus nutrition index is unlike the P concentration in the biomass of wheat correlated with the M3 concentration in the soil and the M3 extractable P concentration in the soil [54].

The differences of the macronutrient uptake between the soil tillage treatments resulted mostly from the changes in the grain and straw yields and less by differences in concentrations. Additionally, for oat and pea in intercrops, the yield affected macronutrient uptake most, whereas concentration changes due to mixing ration and fertilization had lower effects [38]. For Luvisol in Canada, no effect of the differences of the P and K stratification between the conventional and zero tillage on the uptake of P and K by wheat grain were reported [55].

## 5. Conclusions

Soil tillage affects element concentration at 0–5 cm soil depth with the highest P concentration in NT (and also of M3 extractable P in CTd) and the highest K concentration in NT and both conservation tillage systems at 0–10 cm soil depth than in MP after 15 years, whereas the distribution of S, Ca and Mg is not affected by the tillage systems. Both extractants, AA and M3, show the distribution pattern of the nutrients in the soil profile but M3 showed higher differences. However, AA could also serve as a simple extractant for the fast screening of macronutrients in the soil.

## Figures and Tables

**Table 1:** Acetic acid extractable and Mehlich 3 extractable macronutrients phosphorus (P), potassium (K), sulphur (S), calcium (Ca) and magnesium (Mg) in two crop rotations A (pre-crop: winter wheat) and B (pre-crop: sugar beet) after 15 years in Raasdorf (Austria). Mean concentrations over the soil depth of 0–50 cm and over all four soil tillage treatments.

		Acetic Acid		Mehlich 3	
		A	B	A	B
P	(mg kg^−1^)	10.8	13.0	70.6	70.4
K	(mg kg^−1^)	98	97	205	218
S	(mg kg^−1^)	38.4	36.4	45.2	44.8
Ca	(g kg^−1^)	45.4	44.0	17.7	18.8
Mg	(mg kg^−1^)	808	928	417	391

**Table 2:** Winter wheat grain and straw yields and harvest index in 2012. Concentrations, uptake and harvest indices of macronutrients as affected by tillage and crop rotation after 15 years of different tillage treatments.

		Soil Tillage				Crop Rotation		ANOVA	
		MP	NT	CTd	CTs	A	B	ST	CR
**Yields**									
Grain yield	kg ha^−1^	1748 ^c^	2299 ^a^	1952 ^bc^	2073 ^ab^	2735 ^a^	1301 ^b^	**	***
Straw yield	kg ha^−1^	3042 ^c^	3943 ^a^	3341 ^bc^	3548 ^ab^	4868 ^a^	2069 ^b^	*	***
Harvest index	%	37.1	37.2	37.5	37.6	36.0 ^b^	38.6 ^a^		*
**Grain concentration**									
P	mg kg^−1^	2518	3111	3064	2885	3297 ^a^	2493 ^b^		**
K	mg kg^−1^	4160	4164	3799	3992	4169	3888		
S	mg kg^−1^	2318	2456	2703	2460	2592 ^a^	2376 ^b^		*
Ca	mg kg^−1^	446 ^a^	384 ^b^	410 ^ab^	449 ^a^	399 ^b^	445 ^a^	*	**
Mg	mg kg^−1^	1143	1102	1098	1155	1197 ^a^	1052 ^b^		**
**Straw concentration**									
P	mg kg^−1^	249	339	251	218	312 ^a^	217 ^b^		*
K	mg kg^−1^	12,618 ^b^	18,290 ^a^	14,385 ^ab^	13,805 ^b^	14,193	15,357	**	
S	mg kg^−1^	1372	1530	1553	1304	1505	1374		
Ca	mg kg^−1^	3021 ^a^	2332 ^b^	3048 ^a^	2935 ^a^	2732	2936	***	
Mg	mg kg^−1^	781	645	639	800	727	706		
**Grain uptake**									
P	kg ha^−1^	4.61 ^b^	7.56 ^a^	6.16 ^ab^	6.50 ^ab^	9.13 ^a^	3.29 ^b^	*	***
K	kg ha^−1^	7.34 ^b^	9.73 ^a^	7.53 ^b^	8.35 ^ab^	11.40 ^a^	5.07 ^b^	*	***
S	kg ha^−1^	4.07 ^b^	5.83 ^a^	5.24 ^a^	5.27 ^a^	7.12 ^a^	3.08 ^b^	*	***
Ca	kg ha^−1^	0.76 ^b^	0.87 ^ab^	0.79 ^b^	0.91 ^a^	1.09 ^a^	0.57 ^b^	*	***
Mg	kg ha^−1^	2.05 ^c^	2.61 ^a^	2.18 ^bc^	2.46 ^ab^	3.27 ^a^	1.37 ^b^	*	***
**Straw uptake**									
P	kg ha^−1^	0.76 ^b^	1.46 ^a^	0.94 ^ab^	0.86 ^b^	1.55 ^a^	0.46 ^b^	*	***
K	kg ha^−1^	37.56 ^b^	72.72 ^a^	45.26 ^b^	49.45 ^b^	69.94 ^a^	32.55 ^b^	***	***
S	kg ha^−1^	4.24 ^b^	6.28 ^a^	5.20 ^ab^	4.75 ^b^	7.38 ^a^	2.85 ^b^	*	***
Ca	kg ha^−1^	9.19	8.74	10.10	10.17	13.16 ^a^	5.95 ^b^		***
Mg	kg ha^−1^	2.48	2.51	2.03	2.87	3.53 ^a^	1.45 ^b^		***
**Nutrient harvest index**									
P	%	85.0	84.7	87.8	88.6	85.6	87.5		
K	%	15.4	12.1	14.1	15.1	14.6	13.6		
S	%	49.8	48.6	50.9	53.6	49.2	52.3		
Ca	%	8.0	9.0	7.6	8.5	7.8 ^b^	8.7 ^a^		*
Mg	%	46.9	50.3	51.1	46.8	48.6	48.8		

Abbreviations: MP = mouldboard ploughing, NT = no-till, CTd = deep conservation tillage, CTs = shallow conservation tillage, ST = soil tillage, CR = crop rotation, A = crop rotation A (pre-crop: winter wheat), B = crop rotation B (pre-crop: sugar beet). Different letters indicate significant differences between Significance levels: *p* < 0.05 (*), *p* < 0.01 (**), *p* < 0.001 (***). Blank cells indicate no significant results (*p* > 0.05). There were no statistically significant interactions of ST × CR.

## Data Availability

Data are available on request from the corresponding author.
